# Variation and plasticity in life-history traits and fitness of wild *Arabidopsis thaliana* populations are not related to their genotypic and ecological diversity

**DOI:** 10.1186/s12862-024-02246-x

**Published:** 2024-05-03

**Authors:** Raul de la Mata, Almudena Mollá-Morales, Belén Méndez-Vigo, Rafael Torres-Pérez, Juan Carlos Oliveros, Rocío Gómez, Arnald Marcer, Antonio R. Castilla, Magnus Nordborg, Carlos Alonso-Blanco, F. Xavier Picó

**Affiliations:** 1 Departamento de Biología Evolutiva, Estación Biológica de Doñana (EBD), Consejo Superior de Investigaciones Científicas (CSIC), Sevilla, 41092 Spain; 2 Faculty of Forestry, Institute of Dehesa Research (INDEHESA), Universidad de Extremadura, 10600 Plasencia, Spain; 3 Gregor Mendel Institute, Austrian Academy of Sciences, 1030 Vienna, Austria; 4 Departamento de Genética Molecular de Plantas, Centro Nacional de Biotecnología (CNB), Consejo Superior de Investigaciones Científicas (CSIC), 28049 Madrid, Spain; 5 CREAF, Bellaterra (Cerdanyola del Vallès), 08193 Catalonia, Spain; 6 Universitat Autònoma de Barcelona, Bellaterra (Cerdanyola del Vallès), 08193 Catalonia, Spain; 7 Department of Plant Biology, Ecology, and Evolution, College of Arts and Sciences, Oklahoma State University, Stillwater, OK 74078-3031 USA

**Keywords:** *Arabidopsis thaliana*, Common garden experiment, Phenotypic plasticity, Phenotypic variation, Within-population variation

## Abstract

**Background:**

Despite its implications for population dynamics and evolution, the relationship between genetic and phenotypic variation in wild populations remains unclear. Here, we estimated variation and plasticity in life-history traits and fitness of the annual plant *Arabidopsis thaliana* in two common garden experiments that differed in environmental conditions. We used up to 306 maternal inbred lines from six Iberian populations characterized by low and high genotypic (based on whole-genome sequences) and ecological (vegetation type) diversity.

**Results:**

Low and high genotypic and ecological diversity was found in edge and core Iberian environments, respectively. Given that selection is expected to be stronger in edge environments and that ecological diversity may enhance both phenotypic variation and plasticity, we expected genotypic diversity to be positively associated with phenotypic variation and plasticity. However, maternal lines, irrespective of the genotypic and ecological diversity of their population of origin, exhibited a substantial amount of phenotypic variation and plasticity for all traits. Furthermore, all populations harbored maternal lines with canalization (robustness) or sensitivity in response to harsher environmental conditions in one of the two experiments.

**Conclusions:**

Overall, we conclude that the environmental attributes of each population probably determine their genotypic diversity, but all populations maintain substantial phenotypic variation and plasticity for all traits, which represents an asset to endure in changing environments.

**Supplementary Information:**

The online version contains supplementary material available at 10.1186/s12862-024-02246-x.

## Background

Although there are few studies monitoring populations for both long-term demographic and genetic dynamics [1], genetic variation is intuitively considered as a valuable attribute for population viability, particularly when populations are challenged by environmental fluctuations [2]. This view results from the positive relationship between genetic variation and population size [3, 4], both used as proxies for population viability. In particular, it is assumed that a population harboring a large pool of genetic variants will have greater chances of buffering the effects of environmental shifts, whereas populations impoverished genetically may see such buffering ability diminished. As selection acts upon phenotypes, a large pool of genetic variants in a population ought to be translated into a broad spectrum of phenotypes to make the buffering effect of genetic variation effective in changing environments. However, empirical data on the actual relationship between genetic and phenotypic variation in populations reveals that such a relationship is rather weak or inexistent [5–10]. Some explanations include past bottlenecks shaping molecular and quantitative variation in a different manner or differential environmental effects on molecular and quantitative traits [6]. Hence, the relationship between genetic and phenotypic variation and its utility to biologists for assessing population viability probably need to be reappraised.

In this study, we evaluated the relationship between genotypic diversity, i.e. the number and frequency of genotypes in a population, and phenotypic variation in life-history traits in wild populations of the self-fertilizing annual plant *Arabidopsis thaliana*. To increase the odds of studying populations not affected by recent disturbances, we selected populations from Iberian environments that remained undisturbed by land-use changes for several decades [11] and that are known to exist at least since the early 2000s, when we sampled most of them for the first time. Furthermore, the limited dispersal of *A. thaliana* [11–15] ensures that the genetic composition of populations was chiefly driven by mutation-selection balance. Here, we quantified variation in life-history traits and fitness of *A. thaliana* maternal inbred lines (maternal lines hereafter), estimated in common garden experiments over two consecutive years that differed in weather conditions. Hence, we also assessed phenotypic plasticity as another component of phenotypic performance displayed by populations [16–18], which may have important implications for population viability and evolutionary dynamics.

We tested two hypotheses to examine the relationship between genotypic diversity, estimated with whole-genome sequences, and phenotypic variation, estimated in common garden experiments, in *A. thaliana* populations. We took advantage of the fact that our study populations split into two groups: one with low and the other with high genotypic diversity. As populations with low genotypic diversity were located in edge environments, mostly determined by low (e.g. seaside location) and high (e.g. mountain passes) elevations across the species’ Iberian distribution, we expected to detect lower phenotypic variation in these populations as selection is expected to be stronger in edge environments [19, 20]. In particular, *A. thaliana* avoids the hot and dry summers at low Iberian elevations, whereas at high altitudes, the species has to overcome harsh winters. This expectation is supported by previous findings on the effects of elevation gradients on Iberian *A. thaliana*, indicating that edge environments are good predictors of genetic attributes (e.g. lower genetic diversity at higher altitudes; [21]), architectural traits (e.g. larger plant size at bolting at higher altitudes; [22]), fitness-related traits (e.g. weaker seed dormancy and later flowering time at higher altitudes; [23]), and demographic features (e.g. dominance of spring-germinated plants at higher altitudes; [24]). In contrast, populations with higher genotypic diversity in core environments at intermediate elevations could show the opposite trend if selection acted in a more relaxed manner.

In addition, our study populations with low genotypic diversity in edge environments were also characterized by lower ecological diversity, determined by the diversity of major vegetation types, than populations with high genotypic diversity in core environments. Thus, we also hypothesized that differences in the ecological diversity of populations promoted lower and higher phenotypic variation in populations with low and high genotypic diversity, respectively. On top of the effects of fine-scale selection on fitness-related traits in heterogeneous environments [25–29], which promote within-population phenotypic variation, phenotypic plasticity has also been seen to be enhanced in environments with higher levels of ecological heterogeneity [30–32]. In addition, environmental maternal effects may also provide transgenerational adaptive plasticity in plants [33–36]. Overall, we predicted that core populations with higher genotypic and ecological diversity exhibited, not only higher phenotypic variation, but higher phenotypic plasticity than those in edge environments.

Here, we asked (*i*) what is the pattern of phenotypic variation and plasticity in life-history traits and fitness estimated in common garden experiments of *A. thaliana* populations differing in genotypic and ecological diversity? And (*ii*) what are the ecological and genetic drivers of phenotypic variation and plasticity in this set of populations representing core and edge Iberian environments? We discuss the results in the context of the intertwined relationship between genetic and phenotypic variation to broaden our comprehension of the evolutionary dynamics of natural *A. thaliana* populations and their viability in changing environments.

## Methods

### Source populations and sampling

**Fig. 1:**
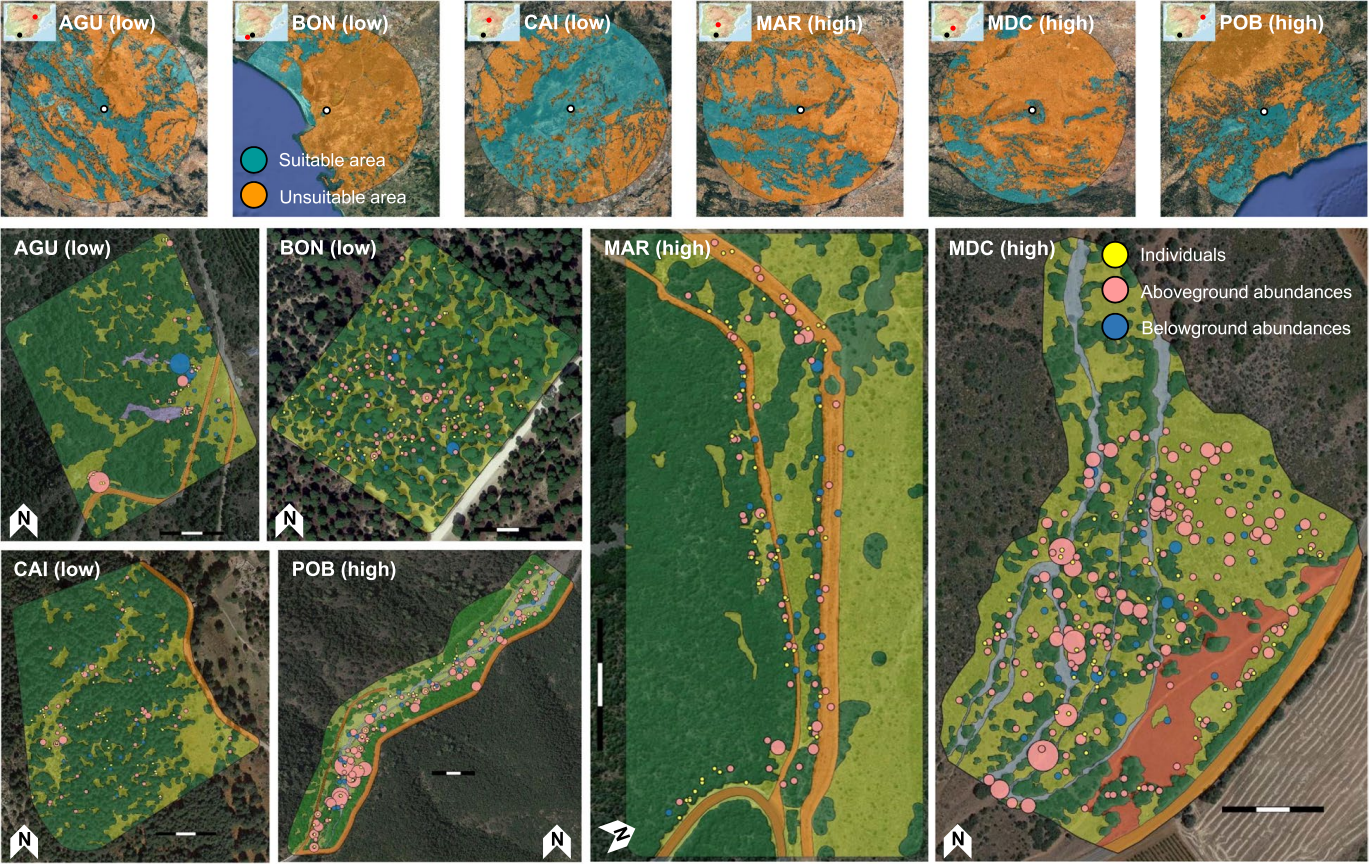
Geographic location and vegetation type of *A. thaliana* populations with low (AGU, BON, and CAI) and high (MAR, MDC, and POB) genotypic and ecological diversity in edge and core environments, respectively. Upper panels depict the regional-scale proportion of suitable and unsuitable area (50 km radius) around each population estimated by pooling vegetation types with *A. thaliana* occurrences using the CORINE Land Cover 2000 database. Population maps are digitized aerial orthophotographs indicating vegetation types: forests (dark green), scrubland (light green), dry riverbeds (blue), boulders (grey), paths, and roads (orange). The location of sampled individuals for seeds and spots to estimate above and belowground abundance is indicated. Dot size is proportional to abundance. Scale bars indicate 100 m

F.X. Picó, R. Gómez and C. Alonso-Blanco collected seeds from all individuals used in this study. It must be noted that *A. thaliana* is a common plant species not categorized as protected or endangered in any species list of the Convention on the Trade in Endangered Species of Wild Fauna and Flora. We selected six populations from the Iberian collection of *A. thaliana* populations [11, 37–41] encompassing some of the environments where the species thrives across the region (Fig. 1 and Fig. S1). From a climatic viewpoint, populations represented mountain (AGU and CAI), continental (MAR and MDC) and coastal (BON and POB) Mediterranean climates. It must be noted that BON and POB, although both under a maritime influence, strongly differ in geographical location, altitude and distance to the coastline (Fig. 1 and Fig. S1). BON and CAI were the warmest and coolest populations, whereas MDC and CAI were the driest and the wettest populations, respectively (Fig. S1). Habitats included seaside stone pine (*Pinus pinea* L.) forests on sandy soils (BON), mixed forests dominated by oak species (POB), sclerophyllous scrublands with holm oak (*Quercus ilex* L.) trees (AGU, MAR and MDC), and mountainous Scots pine (*Pinus sylvestris* L.) forests (CAI) (Fig. S1).

Between early March and mid-June 2017, we haphazardly collected seeds from 50–60 individuals per population (area: 5.9–7.4 ha; Fig. 1 and Fig. S1). In the case of BON, we had to collect seed again in March 2018 to complete the panel of individuals. Maternal lines from BON generated from the 2018 sampling (see below) could not be used in analyses including the first experiment because they had not completed the after-ripening, affecting their performance in the first experiment (Supplementary Methods). We searched *A. thaliana* by covering sections within each population following a non-fixed zigzag path until completing the whole area [15]. In spring 2017 and 2018, we estimated aboveground abundance by haphazardly selecting 21–182 spots per population. (Fig. 1). In each spot, we counted *A. thaliana* individuals (all flowering and fruiting) in circular areas (5 m radius) with a hand tally counter.

By midsummer, *A. thaliana* seeds disperse and the first cm of soil are completely dry in all populations. Thus, in July 2018, we estimated belowground abundance (seed bank) by selecting 24 spots in each population based on the map of abundances (Fig. 1). In each spot, we collected soil from the upper soil layer (0–5 cm depth) at several points in circular areas (5 m radius). We pooled, sieved (to 2 mm) and stored dry soil samples from each spot in plastic tubes (225 ml). In October 2019, we conducted a germination experiment to estimate belowground *A. thaliana* abundance. We spread out soil samples (6 populations × 24 samples/population = 144 soil samples) on plastic trays (60 × 40 × 7 cm^3^) with moistened filter paper at the bottom. Plastic trays, covered with crystal-clear sealing film, were placed in a FITOCLIMA-10.000-EH growth chamber (ARALAB, Rio de Mouro, PT) at the Estación Biológica de Doñana (Sevilla, ES). We applied a cold treatment (4ºC in darkness over 2 weeks) to break seed dormancy, followed by a light treatment (20ºC in light over 1 week followed by 16 h of light/8 h of darkness over 3 additional weeks) to promote germination. We took pictures of each tray weekly and counted seedlings with the software ImageJ2 [42]. 

We used GPS (Garmin International, Inc., Olathe, US; positional error: 4 m) to record coordinates of individuals and spots. We calculated the proportion of each vegetation type in a circular area around the GPS coordinate of each individual (1 m radius) and spot (5 m radius) using a digitized aerial orthophotograph of each population [11, 15] from which vegetation type distributions were previously estimated (Fig. 1). The maximum membership proportion (range among populations = 0.91–0.98 and 0.68–0.79 for individuals and spots, respectively) was used to assign a vegetation type to each individual and spot. We computed the Shannon–Wiener index to estimate vegetation type diversity for individuals and spots.

### Common garden experiments

Over fall and winter 2017–2018, we multiplied field-collected seeds via single-seed descent in a glasshouse at the Centro Nacional de Biotecnología (Madrid, ES). Overall, we obtained seeds from 55, 14, 50, 50, 53 and 53 maternal lines from AGU, BON, CAI, MAR, MDC and POB, respectively. In summer 2018, we multiplied seeds from 31 additional maternal lines collected in BON in March 2018 (Supplementary Methods), totaling 306 maternal lines. These materials are publicly available through the Nottingham Arabidopsis Stock Centre (NASC). We used multiplied seeds, minimizing environmental and maternal effects, to undertake common garden experiments at the Botanical Garden of Sierra de Grazalema Natural Park (36.46ºN, 5.30ºW; 329 m a.s.l.), over two years (2018–2019 and 2019–2020) to estimate variation in life-history traits and fitness [15, 39, 43, 44].

In summer 2018 and 2019, we prepared eight batches (replicates) of 60 seeds each per maternal line (306 maternal lines × 8 batches/maternal line × 60 seeds/batch = 146 880 seeds per experiment). We established experiments on the same date (November 15) and on the same exact stands to set up eight blocks (Fig. S2). We sowed seeds from each replicate and maternal line in square plastic pots (12 × 12 × 12 cm^3^) filled with standard soil mixture (Cejudo Baena S.L., Utrera, ES) and randomly placed one replicate per maternal line in each block (306 maternal lines × 8 replicates/maternal line = 2 448 pots per experiment).

We estimated recruitment as the maximum proportion of seedlings in each pot, recorded within the first 2–3 weeks after sowing. We estimated flowering time as the number of days between sowing and flowering dates, given by the date most plants in the pot (full-sibs with homogeneous behavior) had the first flower open. Paper bags were used to collect all plants when flowering finished and before fruit dehiscence. We recorded the number of plants per pot and the number of fruits per plant. Fecundity was estimated as the number of seeds per plant with a non-linear function relating the number of fruits per plant with the number of seeds per fruit [43]. We estimated survival as the proportion of plants relative to the maximum number of seedlings recorded. We estimated fitness (survival × fecundity) as the mean number of expected seeds per plant.

The national lockdown due to the coronavirus pandemic prevented us from monitoring the second experiment between mid-March and mid-June 2020. Nonetheless, we were able to impute flowering dates to those replicates of maternal lines with missing data. Practically all replicates of maternal lines from BON, MAR and MDC had complete flowering dates before the lockdown (Table S1), whereas 11, 21 and 38% of replicates of maternal lines from POB, CAI and AGU, respectively, had missing flowering dates. Imputation was feasible because we used the very same stands to set up the blocks in both experiments and because flowering dates were systematically earlier in the second experiment (Supplementary Methods; Table S1).

We recorded daily temperature and precipitation between establishment (November 15) and termination (April 15) dates in both experiments (Fig. S3). We obtained daily minimum and maximum temperature with HOBO Pendant UA-002–08 temperature loggers (Onset Computer Corporation, Inc., Bourne, US) and daily precipitation from data provided by the automatic meteorological station of the experimental facility.

### Genetic characterization

We grew all *A. thaliana* maternal lines in a greenhouse at the Gregor Mendel Institute (Vienna Biocenter, Vienna, AT) to generate whole-genome shot-gun sequences (Supplementary Methods). Sequences were analyzed by the Service of Bioinformatics for Genomics and Proteomics at the Centro Nacional de Biotecnología (Madrid, ES). We generated a final VCF file containing 2 798 036 non-singleton nuclear SNPs genotyped in 298 of 306 maternal lines (Supplementary Methods). We generated a pairwise matrix of allele differences from this VCF file, which was used to identify pairs of maternal lines with nearly identical genotypes. Pairs of samples with genetic distances lower than 0.001 were considered as carrying the same genotype, because this was the genotyping error that we estimated by sequencing twice five MDC samples and in agreement with error sequencing rates described for Illumina short reads [45].

For each population, we assigned maternal lines to genotypes and computed the Shannon–Wiener index to estimate genotypic diversity. We did not detect shared genotypes among populations. As heterozygous SNPs were rescored to the major frequency allele to obtain the final VCF file (Supplementary Methods), we estimated mean observed heterozygosity per population with 12 neutral nuclear microsatellites genotyped in 282 of 306 maternal lines. To this end, we pooled DNA from six full-sibs from each maternal line grown in a growth chamber at the Estación Biológica de Doñana (Sevilla, ES) to extract DNA (details on DNA extraction, marker genotyping and genotyping error as in [37]).

### Statistical analyses

We used linear mixed models to test the effect of group (fixed factor; populations with low and high genotypic and ecological diversity), experiment (fixed factor; 2018–2019 and 2019–2020), population nested within group (random factor; AGU, BON and CAI for the low diversity group in edge environments, and MAR, MDC and POB for the high diversity group in core environments), and maternal line (random factor) nested within population on life-history traits (recruitment, flowering time, survival and fecundity) and fitness in *A. thaliana* estimated in common garden experiments. The random interactions of experiment with population and maternal line were excluded from the model due to lack of convergence. As the model did not converge with survival, we replaced survival by the number of fruiting plants, used to estimate survival and closely related to recruitment (*R*^*2*^ = 0.71). We estimated variance components of random factors using the restricted maximum likelihood (REML) method. Significance of factors were tested using log-likelihood ratio tests and differences between population pairs were tested with Tukey post-hoc tests. We inspected variances of data, the existence of outliers and model residuals to check that the major assumptions of the analyses were acceptable. We fitted the linear mixed model using the MIXED procedure of SAS v.9.4 [46].

For each population and experiment, we estimated the correlation between pairs of traits with Dutilleul’s *t*-test, including the spatial autocorrelation of data, with the software SAM v.4.0 [47]. To estimate the contribution of genotypic variance to phenotypic variance of life-history traits and fitness in *A. thaliana* maternal lines from populations with low and high diversity in edge and core environments, respectively, estimated in the common garden experiments, we estimated broad-sense heritability (*H*^*2*^) values as *H*^*2*^ = *V*_*G*_/(*V*_*G*_ + *V*_*E*_), where *V*_*G*_ is the among-maternal line variance component and *V*_*E*_ is the residual variance [48]. We estimated all variance components and their 95% confidence intervals using the *remlVCA* and *VCAinference* functions of the R package VCA v.1.4.3. (https://cran.rproject.org/web/packages/VCA/index.html). 

We estimated phenotypic plasticity for life-history traits and fitness for each maternal line by computing the relative distance plasticity index (RDPI) [49], which ranges between 0 (no plasticity or canalization) and 1 (maximal plasticity or sensitivity), between the two common garden experiments. We tested the fixed effect of group (edge and core environments with low and high diversity, respectively) and the random effect of population nested within group on phenotypic plasticity of life-history traits and fitness with linear mixed models with the *lmer* function of the the R package lme4 [50] using maternal lines as replicates. We calculated the number of maternal lines from each population that significantly differed in life-history traits between experiments using Student’s *t*-tests. We also analyzed the distribution of maternal lines with low and high phenotypic plasticity among populations and traits with G-tests. To explore whether plasticity in recruitment and flowering time was related to fitness, we correlated plasticity distance matrices for these fitness components with fitness distance matrices with Mantel tests with PASSaGE v.2 [51].

We identified the drivers of variation in life-history traits and fitness of *A. thaliana* populations harboring low and high diversity in edge and core environments, respectively, by correlating pairwise phenotypic distance matrices with geographic, genetic and suitability distance matrices using Mantel tests. For each population, we obtained the geographic distance matrix as a matrix of Euclidian distances using GPS coordinates of sampled individuals. We generated the genetic distance matrix as a matrix of pairwise allelic differences among genotyped maternal lines with whole-genome sequences. We estimated a suitability distance matrix by using aboveground abundances, as spots with higher abundances are likely to be more suitable for *A. thaliana*. To do that, we generated a Voronoi diagram, in which each cell contained one spot to estimate aboveground abundance within each population (Fig. S4), with the function *voronoi* in the R package terra (https://cran.r-project.org/web/packages/terra/index.html). A suitability value to each sampled individual was assigned with the function *st_intersection* of the R package sf [52]. We performed all Mantel tests with standardized variables (subtracting the mean and scaling the variance) and estimated significances with 1000 permutations. Given the number of Mantel tests conducted, we only considered as significant those relationships with *P* < 0.01 (tests with *P* < 0.05 were few and with very low coefficients).

Finally, to identify the effects of selection on *A. thaliana* maternal lines from populations with low and high diversity in edge and core environments, respectively, estimated in the common garden experiments, we estimated linear and quadratic selection gradients (β and γ) and selection differentials (*s* and *C*) for recruitment and flowering time using traditional least squares-based regressions with fitness using the R package gsg [53]. We estimated all parameters from full models with linear and quadratic effects using standardized variables. 

## Results

### Genetic and ecological attributes of study populations

The analysis of whole-genome sequences showed that the six *A. thaliana* study populations split into populations with low (AGU, BON and CAI) and high (MAR, MDC and POB) genotypic diversity in edge and core environments, respectively (Table 1). Populations with low genotypic diversity in edge environments had about one-fourth of maternal lines with different genotypes, of which about 50% or less were unique (represented by one maternal line only). In contrast, about four-fifths of maternal lines from populations with high genotypic diversity in core environments exhibited different genotypes, of which more than 80% were unique. Thus, the Shannon–Wiener index was lower in populations with low (range = 1.67–2.09) than high (range = 3.36–3.74) genotypic diversity. In addition, observed heterozygosity was 1–2 orders of magnitude lower in populations with low than high genotypic diversity in edge and core environments, respectively (Table 1).

**Table 1:** Ecological and genetic attributes of six *A. thaliana* populations. Ecological attributes encompass the percentage of vegetation types, including forest, scrubland and others (dry riverbeds, boulders, paths, and roads) across study areas. Estimates of above and belowground abundance are given by the mean (± SE) number of *A. thaliana* individuals observed or estimated in areas of ca. 20 m^2^. Genetic attributes include observed heterozygosity *H*_*O*_ (± SE) based on 12 nuclear microsatellites (SSR), and the proportion of different and unique genotypes (represented by one individual only) estimated with whole-genome sequences (WGS). Genetic and ecological attributes allow the distinction of populations harboring low (AGU, BON, and CAI) and high (MAR, MDC, and POB) genotypic and ecological diversity in edge and core environments, respectively

	Ecological attributes					Genetic attributes		
	Vegetation type			Abundance		SSR	Genotypes (WGS)	
Population	Forest	Scrubland	Others	Aboveground	Belowground	*H*_*O*_	Different	Unique
AGU (low)	66.8	27.6	5.6	121.9 ± 42.6	5.4 ± 2.7	0.009 ± 0.003	0.27	0.50
BON (low)	66.0	34.0	0.0	30.8 ± 3.8	8.6 ± 4.1	0.004 ± 0.003	0.20	0.33
CAI (low)	68.4	28.4	3.2	50.4 ± 7.6	0.6 ± 0.2	0.006 ± 0.003	0.24	0.50
MAR (high)	54.8	35.0	10.2	51.6 ± 7.1	2.2 ± 0.8	0.109 ± 0.010	0.73	0.82
MDC (high)	30.6	48.4	21.0	98.0 ± 7.0	5.9 ± 1.0	0.017 ± 0.007	0.83	0.87
POB (high)	47.1	33.4	19.5	121.8 ± 13.0	1.9 ± 0.5	0.038 ± 0.008	0.88	0.87

From an ecological viewpoint, *A. thaliana* populations were located in contrasting environments chiefly defined by geography (Fig. 1), thereby affecting their climatic and ecological features (Fig. S1). The diversity of vegetation types within populations occupied by *A. thaliana* was the most remarkable difference between the two groups of populations (Table 1). Populations with low genotypic diversity in edge environments were massively dominated by forest and scrubland, whereas populations with high genotypic diversity in core environments encompassed a greater diversity of vegetation types, including forest, scrubland and openings made by dry riverbeds, boulders, or paths. The Shannon–Wiener index for vegetation type diversity of sampled individuals and spots to estimate abundances captured such differences: individuals and spots exhibited lower vegetation type diversity in populations with low (range = 0.54–0.76 and 0.66–0.85 for individuals and spots, respectively) than high (0.82–1.07 and 0.84–1.06 for individuals and spots, respectively) genotypic diversity in edge and core environments, respectively.

Above and belowground *A. thaliana* abundances (Fig. 1 and Fig. S4) did not differentiate populations with low and high diversity in edge and core environments, respectively. For example, the two populations with the highest mean aboveground abundance, but also with the highest variance, were AGU (low diversity) and POB (high diversity) (Table 1). The same applied for belowground abundance, as BON and AGU (both with low diversity) and MDC (high diversity) exhibited the highest mean belowground abundances (Table 1). Overall, there was no relationship between mean above and belowground abundances. For example, the two populations with the highest mean aboveground abundances (AGU and POB) had low and high mean belowground abundances (Table 1). Finally, BON was the population with the lowest mean aboveground abundances and had the highest mean belowground abundance, whereas CAI had the second lowest mean aboveground abundance and a nearly inexistent mean belowground abundance (Table 1).

### Variation in life-history traits and fitness

We estimated the genetic component of variation in life-history traits and fitness of *A. thaliana* maternal lines from populations with low and high diversity in edge and core environments, respectively, in two common garden experiments. Weather conditions differed between experiments (Fig. S3). In particular, average minimum temperatures over the course of the first experiment were cooler (mean ± SD = 4.89 ± 2.85 ºC; range = -1.66–11.87 ºC) than those of the second experiment (6.44 ± 3.05 ºC; 1.46–14.65 ºC), although average maximum temperatures were similar in both experiments (23.47 ± 6.48 ºC and 23.14 ± 5.43 ºC for the first and second experiment, respectively). The second experiment was rainier (total rainfall over the experiment = 792.65 mm) than the first one (502.55 mm), although the amount of rainfall was more evenly distributed in the first (CV of rainfall over the experiment = 270.05%) than in the second (CV = 344.91%) experiment. On top of warmer temperatures, a heavy rain recorded in one day (180 mm on December 20, 2019) and a dry early spring with a delayed rainfall almost at the end of the experiment, affected the development of *A. thaliana* in the second experiment (Fig. S3), as shown by the number of maternal lines that did not complete the life cycle in the second experiment (range among populations = 2–15 maternal lines in POB and CAI, respectively).

Linear mixed models indicated that the two groups of populations with low and high diversity in edge and core environments, respectively, did not differ for recruitment, flowering time and survival (Table 2). In contrast, they significantly differed for fecundity and fitness (Table 2). Maternal lines from populations with low diversity produced less seeds per plant (mean ± SE = 111.25 ± 4.06 seeds per plant) than those with high diversity (144.11 ± 2.98 seeds per plant). Likewise, maternal lines from populations with low diversity exhibited lower fitness (78.98 ± 2.66 expected seeds per plant) than those with high diversity (102.87 ± 2.30 expected seeds per plant). In addition, all traits significantly differed between experiments (Table 2), although with different patterns. In particular, maternal lines showed higher recruitment (0.56 ± 0.01 and 0.47 ± 0.01 for the first and second experiment), later flowering time (115.81 ± 0.43 and 110.88 ± 0.55 days for the first and second experiment), higher survival (0.87 ± 0.01 and 0.57 ± 0.01 for the first and second experiment), and higher fitness (97.93 ± 2.19 and 86.26 ± 2.85 expected seeds/plant) in the first than in the second experiment. In contrast, maternal lines showed lower fecundity in the first (110.62 ± 2.30 seeds/plant) than in the second experiment (147.97 ± 4.27 seeds/plant).

**Table 2:** Summary of the linear mixed model testing the effect of group (populations with low and high genotypic and ecological diversity in edge and core environments, respectively), experiment (2018–2019 and 2019–2020), population nested within group (AGU, BON, and CAI; MAR, MDC, and POB), and maternal family nested within population on life-history traits and fitness in *A. thaliana*. *F*-values and variance components (± SE) are given for fixed and random factors, respectively. Asterisks indicate significance: ***P < 0.001, **P < 0.01, *P < 0.05, *ns*; non-significant

Trait	Group	Experiment	Population	Maternal family
	*F*-value	*F*-value	Variance comp	Variance comp
Recruitment	0.01 ns	354.24 ***	41.08 ± 30.20 ***	23.62 ± 2.97 ***
Flowering time	0.20 ns	524.58 ***	76.26 ± 54.44 ***	15.80 ± 1.54 ***
Survival	0.02 ns	1213.10 ***	38.74 ± 28.24 ***	14.31 ± 2.33 ***
Fecundity	21.09 *	121.18 ***	48.14 ± 55.16 ns	239.80 ± 94.60 **
Fitness	51.66 ***	16.90 ***	0.00 ± 0.00 ns	185.30 ± 62.19 ***

**Fig. 2:**
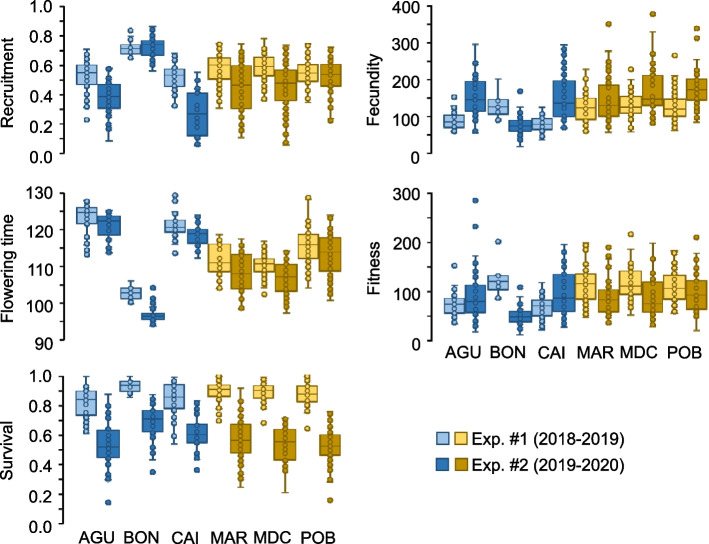
Summary statistics for life-history traits and fitness of *A. thaliana* populations with low (AGU, BON, and CAI) and high (MAR, MDC, and POB) genotypic and ecological diversity in edge and core environments, respectively, estimated in two common garden experiments. For each population and experiment, boxes show the lower and upper quartiles, whiskers are drawn down to the 10th percentile and up to the 90th, the line is the median of observations, and dots indicate data points. Recruitment and survival are proportions, flowering time is given in days, and fecundity and fitness are given in seeds per plant and expected seeds per plant, respectively. Light and dark tones correspond to the first and second common garden experiments, respectively, whereas blue and ochre colors refer to populations from low and high diversity groups in edge and core environments, respectively

Populations within groups (low and high diversity in edge and core environments, respectively) significantly differed for recruitment, flowering time and survival (Table 2 and Fig. 2), but with a different pattern. For example, for populations with low diversity in edge environments, BON was the only population significantly different from AGU and CAI for these three traits (|*t*|> 3.49, *P* < 0.007 in all cases; Tukey post-hoc test), whereas for populations with high diversity in core environments, POB was the only population significantly different from MAR and MDC for flowering time only (|*t*|> 5.19, *P* < 0.0001 in both cases; Tukey post-hoc test). Coefficients of variation for all traits also reflected these patterns of variation among populations within each diversity group (Table S2). The minimum and maximum coefficients of variation tended to be found among populations with low diversity (Table S2). Finally, maternal lines within each population exhibited significant differences for all traits (Table 2). Broad-sense heritability values were mostly significantly different from zero for recruitment and flowering time, whereas those for survival, fecundity and fitness tended to be indistinguishable from zero (Table S3), particularly in the first experiment.

The pattern of pairwise correlation between traits for each population exhibited important differences between experiments (Table S4). In particular, significant correlations decreased in the second experiment (16 correlations) with respect to those detected in the first experiment (23 correlations). In general, populations with low diversity in edge environments showed a lower number of significant correlations between traits (17 correlations) than populations with high diversity in core environments (22 correlations). Overall, when significant, we observed a general trend: early flowering correlated with higher recruitment, higher survival, higher fecundity, and consequently higher fitness.

### Phenotypic plasticity in life-history traits and fitness

**Fig. 3:**
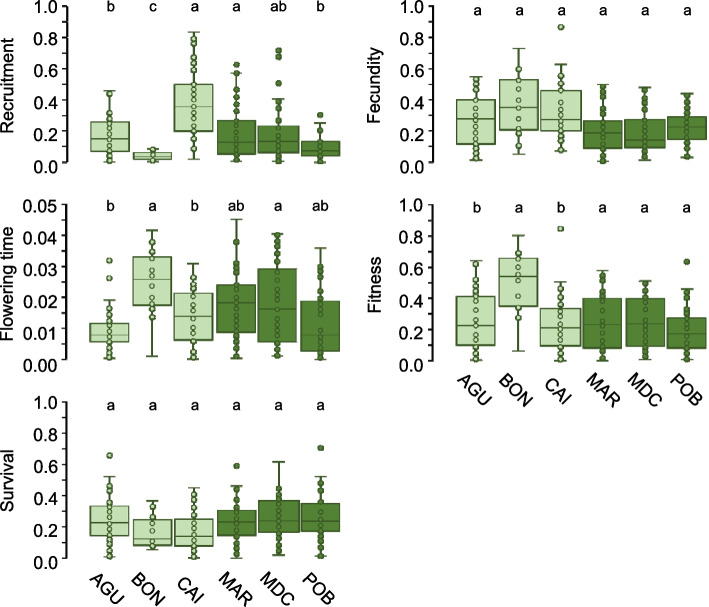
Summary statistics for plasticity of life-history traits and fitness of *A. thaliana* populations with low (AGU, BON, and CAI) and high (MAR, MDC, and POB) genotypic and ecological diversity in edge and core environments, respectively, estimated in two common garden experiments. For each population, boxes show the lower and upper quartiles, whiskers are drawn down to the 10th percentile and up to the 90th, the line is the median of observations, and dots indicate data points. For each trait, different letters indicate significance differences among populations within each diversity group (*P* < 0.05; Tukey post-hoc tests). Light and dark green correspond to populations from low and high diversity groups in edge and core environments, respectively

Given the significant between-experiment differences for all traits, we estimated phenotypic plasticity between experiments for all maternal lines with complete data for all traits. Linear mixed models indicated that there were significant differences in phenotypic plasticity for recruitment, flowering time and fitness (λ_LR_ > 11.84, *P* < 0.004 in all cases; log-likelihood ratio tests), and non-significant for survival and fecundity (λ_LR_ < 0.01, *P* > 0.13 in all cases; log-likelihood ratio tests) (Fig. 3). Differences in phenotypic plasticity among populations within groups (low and high diversity in edge and core environments, respectively) tended to be more pronounced in populations with low than high diversity, particularly for recruitment, flowering time and fitness. Nevertheless, coefficients of variation for plasticity were rather similar among populations with minimum and maximum values detected in populations with either low or high diversity in edge and core environments, respectively (Table S2). The relationships between plasticity in fitness and plasticity in recruitment and flowering time were not significant in any population and experiment.

All populations showed maternal lines with low and high plasticity, as indicated by the number of maternal lines per population falling in the upper and lower 10th percentiles of phenotypic variation (Table 3). The number of maternal lines across populations and traits was unevenly distributed for the upper 10th percentile of phenotypic plasticity (χ^2^ = 69.36, *P* < 0.0001; G-test), but not for the lower 10th percentile (χ^2^ = 31.06, *P* = 0.054; G-test). For the upper 10th percentile, we found a high number of plastic maternal lines for recruitment in CAI (low diversity) and for flowering time in MDC (high diversity), and a lack of plastic maternal lines for recruitment and survival in BON (low diversity) and for recruitment and fecundity in POB (high diversity).

**Table 3:** Number of total maternal lines in the upper and lower 10th percentile of plasticity for life-history traits (recruitment, flowering time, survival and fecundity) and fitness (survival × fecundity) of six *A. thaliana* populations, three with low (AGU, BON, and CAI) and three with high (MAR, MDC, and POB) genotypic and ecological diversity in edge and core environments, respectively, estimated from the first (2018–2019) and second (2019–2020) experiments conducted in the same common garden facility

	AGU (low; 45)		BON (low; 14)		CAI (low; 35)		MAR (high; 45)		MDC (high; 48)		POB (high; 48)	
Trait	Upper	Lower	Upper	Lower	Upper	Lower	Upper	Lower	Upper	Lower	Upper	Lower
Recruitment	2	4	0	3	13	0	4	3	5	5	0	9
Flowering	1	3	4	1	1	6	5	2	11	3	2	9
Survival	7	5	0	2	2	7	4	5	5	2	6	3
Fecundity	7	4	5	1	8	0	3	9	1	6	0	4
Fitness	6	4	9	0	2	4	3	7	3	4	1	5

### Drivers of phenotypic variation and plasticity in life-history traits and fitness

We explored the effects of ecological and genetic drivers of variation on life-history traits and fitness of maternal lines from populations with low and high diversity in edge and core environments, respectively (Table 4). The results indicated that, when significant, genetic similarity (14 cases across all populations, except BON) was more important than geographic similarity (4 cases in CAI) for trait differentiation. We detected more significant relationships in the first (11 cases in flowering time, fecundity and fitness) than in the second (7 cases time in flowering and survival) experiment. Flowering time was the trait with the highest number of significant correlations (11 cases), followed by fitness (3 cases), fecundity (3 cases) and survival (1 case).

**Table 4:** Correlation coefficients from Mantel tests comparing phenotypic distance with geographic (Geo) and genetic (Gen) distances for life-history traits (recruitment, flowering time, survival, and fecundity) and fitness (survival × fecundity) of six *A. thaliana* populations, three with low (AGU, BON, and CAI) and three with high (MAR, MDC, and POB) genotypic and ecological diversity in edge and core environments, respectively, estimated from the first (2018–2019) and second (2019–2020) experiments conducted in the same common garden facility. Sample size is given in parenthesis. Significant coefficients are indicated in boldface. Significance:  *** , *P* < 0.0001;  ** , *P* < 0.01; *ns*; non-significant

2018–2019	AGU (low; 47)		BON (low; 14)		CAI (low; 50)		MAR (high; 48)		MDC (high; 53)		POB (high; 50)	
Trait	Geo	Gen	Geo	Gen	Geo	Gen	Geo	Gen	Geo	Gen	Geo	Gen
Recruitment	0.04 *ns*	0.05 *ns*	-0.07 *ns*	0.08 *ns*	-0.02 *ns*	0.01 *ns*	-0.01 *ns*	-0.03 *ns*	0.13 *ns*	-0.03 *ns*	0.08 *ns*	0.04 *ns*
Flowering	-0.01 *ns*	**0.34** ***	0.08 *ns*	0.15 *ns*	**0.38** ***	**0.51** ***	0.03 *ns*	**0.16** **	0.07 *ns*	0.14 *ns*	-0.06 *ns*	**0.22** **
Survival	-0.03 *ns*	0.04 *ns*	0.05 *ns*	0.22 *ns*	0.02 *ns*	0.13 *ns*	-0.01 *ns*	0.01 *ns*	0.06 *ns*	-0.01 *ns*	-0.05 *ns*	0.03 *ns*
Fecundity	0.03 *ns*	0.09 *ns*	0.17 *ns*	0.19 *ns*	**0.15** **	0.09 *ns*	0.05 *ns*	**0.23** **	0.05 *ns*	**0.32** **	0.01 *ns*	-0.01 *ns*
Fitness	-0.02 *ns*	0.07 *ns*	0.12 *ns*	0.26 *ns*	**0.15** **	0.07 *ns*	0.05 *ns*	**0.28** **	0.04 *ns*	**0.31** **	0.03 *ns*	0.04 *ns*
2019–2020	AGU (low; 45)		BON (low; 42)		CAI (low; 35)		MAR (high; 45)		MDC (high; 48)		POB (high; 48)	
Trait	Geo	Gen	Geo	Gen	Geo	Gen	Geo	Gen	Geo	Gen	Geo	Gen
Recruitment	0.04 *ns*	0.01 *ns*	0.01 *ns*	-0.02 *ns*	0.01 *ns*	0.04 *ns*	0.06 *ns*	0.10 *ns*	-0.01 *ns*	-0.05 *ns*	-0.03 *ns*	0.03 *ns*
Flowering	-0.02 *ns*	**0.32** ***	0.08 *ns*	0.01 *ns*	**0.37** ***	**0.38** ***	0.10 *ns*	**0.16** **	-0.01 *ns*	**0.21** **	-0.04 *ns*	**0.19** **
Survival	0.07 *ns*	0.05 *ns*	-0.01 *ns*	0.03 *ns*	0.02 *ns*	0.16 *ns*	-0.01 *ns*	0.01 *ns*	-0.10 *ns*	-0.04 *ns*	-0.10 *ns*	**0.10** **
Fecundity	0.05 *ns*	-0.01 *ns*	0.12 *ns*	-0.01 *ns*	-0.02 *ns*	-0.10 *ns*	0.03 *ns*	-0.03 *ns*	0.05 *ns*	0.12 *ns*	-0.06 *ns*	-0.01 *ns*
Fitness	0.06 *ns*	0.01 *ns*	0.12 *ns*	-0.02 *ns*	0.01 *ns*	-0.06 *ns*	0.01 *ns*	0.01 *ns*	0.06 *ns*	0.26 *ns*	-0.08 *ns*	0.05 *ns*

Correlations between phenotypic distance and suitability distance were only significant for flowering time in CAI (low diversity; *r* = 0.38, *P* = 0.001; second experiment; Mantel test) and POB (high diversity; *r* = 0.48, *P* = 0.001; first experiment; Mantel test). Correlations between phenotypic plasticity distance and geographic distance were only significant for recruitment in AGU (low diversity; *r* = 0.30, *P* = 0.001; Mantel test) and fitness in BON (low diversity; *r* = 0.35, *P* = 0.007; Mantel test), whereas none of the correlations between phenotypic plasticity distance and genetic or suitability distance were significant (range of |*r*|= 0.00–0.29, *P* > 0.011 in all cases; Mantel tests).

### Selection analysis

We estimated selection gradients and selection differentials for recruitment and flowering time to examine how natural selection acted upon *A. thaliana* phenotypes in the common garden experiments. Linear selection gradients were significant and negative for flowering time in the first experiment in all populations (Table 5 and Table S5), indicating that selection favored *A. thaliana* individuals with earlier flowering in the conditions encountered in the first experiment. In the second experiment, significant linear selection gradients were scarce, weaker and very different from those detected in the first experiment (Table 5 and Table S5). In particular, we found a significant negative linear selection gradient for recruitment in CAI (low diversity), suggesting that selection favored *A. thaliana* individuals with lower recruitment, and a significant positive linear selection gradient for flowering time in POB (high diversity), suggesting that selection favored *A. thaliana* individuals with later flowering.

**Table 5:** Linear selection gradients (*β*) for recruitment and flowering time for six *A. thaliana* populations, three with low (AGU, BON, and CAI) and three with high (MAR, MDC, and POB) genotypic and ecological diversity in edge and core environments, respectively, estimated from the first (2018–2019) and second (2019–2020) experiments conducted in the same common garden facility. Sample size (*N*) for each population and experiment used in the analysis is indicated. *β*-values for BON in the first experiment could not be estimated due to low sample size (see text for details). See Table S5 for results of the complete analysis. Significance: ***, *P* < 0.0001; **, *P* < 0.01; *, *P* < 0.05; *ns*, non-significant

Population	*N* (1st–2nd)	Recruitment (1st exp.)	Flowering time (1st exp.)	Recruitment (2nd exp.)	Flowering time (2nd exp.)
AGU (low)	55–52	0.028 (0.044) *ns*	-0.219 (0.077) **	-0.115 (0.083) *ns*	0.109 (0.142) *ns*
BON (low)	14–42	–	–	0.108 (0.084) *ns*	-0.079 (0.094) *ns*
CAI (low)	50–35	0.006 (0.045) *ns*	-0.217 (0.057) ***	-0.249 (0.128) *	0.014 (0.143) *ns*
MAR (high)	50–47	0.045 (0.050) *ns*	-0.151 (0.058) *	-0.159 (0.095) *ns*	0.054 (0.096) *ns*
MDC (high)	53–48	0.016 (0.039) *ns*	-0.179 (0.039) ***	0.006 (0.109) *ns*	-0.057 (0.113) *ns*
POB (high)	53–51	-0.005 (0.040) *ns*	-0.216 (0.043) ***	0.029 (0.077) *ns*	0.169 (0.080) *

### Variation in life-history traits and fitness at the maternal line level

**Fig. 4:**
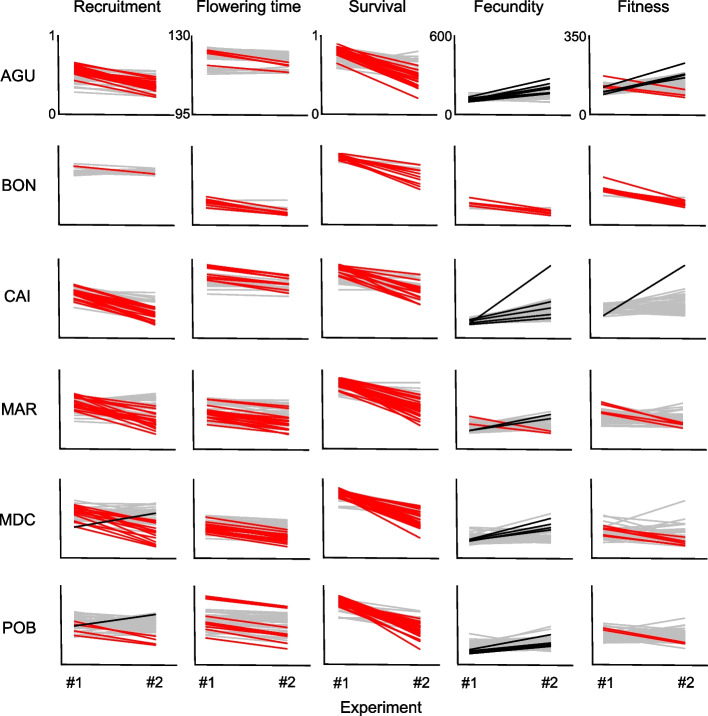
Reaction norms of maternal lines from *A. thaliana* populations with low (AGU, BON, and CAI) and high (MAR, MDC, and POB) genotypic and ecological diversity in edge and core environments, respectively, with significant (red lines: decrease; black lines: increase) and non-significant (grey lines) differences in life-history traits and fitness between the two common garden experiments

We tested the differences between experiments in life-history traits for each maternal line from populations with low and high diversity. The results indicated that all populations, irrespective of their environment type with distinct genotypic and ecological diversity, exhibited maternal lines with significant and non-significant variation between experiments in all traits (Fig. 4). Although this experiment was not conceived to quantify differences among maternal lines within genotypes, we detected maternal lines sharing the same (nearly identical) genotype with significant and non-significant differences between experiments for almost all traits in populations with low and high diversity in edge and core environments, respectively (Fig. S5).

In BON (low diversity), all maternal lines with significant differences between experiments exhibited lower values for all traits in the second experiment (Fig. 4). There were generalized decreases in the second experiment for recruitment, flowering time and survival for all populations, except one individual from MDC and one from POB (both with high diversity) that increased recruitment in the second experiment (Fig. 4). In contrast, fecundity and fitness exhibited further variability (Fig. 4). Maternal lines with significant differences between experiments increased fecundity in the second experiment in two populations with low (AGU and CAI) and high (MDC and POB) diversity. In MAR (high diversity), there were two maternal lines that increased and two that decreased fecundity in the second experiment. In the case of fitness, all maternal lines with significant differences between experiments decreased fitness in the second experiment in all populations with high diversity (Fig. 4). In contrast, in CAI (low diversity) there was one maternal line that increased fitness in the second experiment, and AGU (low diversity) exhibited four and three maternal lines that increased and decreased fitness in the second experiment, respectively (Fig. 4).

The number of maternal lines with significant variation between experiments was unevenly distributed across populations and traits (χ^2^ = 73.13, *P* < 0.0001; G-test). However, there were differences between groups of populations. Populations with low diversity in edge environments significantly differed among them in the distribution of maternal lines with significant variation in traits between experiments, particularly for the comparisons between BON and AGU (χ^2^ = 13.21, *P* = 0.004; G-test), and BON and CAI (χ^2^ = 10.95, *P* = 0.012; G-test). In contrast, all populations with high diversity in core environments did not show significant differences among them in the distribution of maternal lines with significant variation in traits between experiments (χ^2^ < 4.94, *P* > 0.18 in all cases; G-tests).

## Discussion

The amount of genetic and phenotypic variation that a single population can harbor and the relationship between these two structural components of populations are far from being understood due to the intertwined interaction among genetic differences, environmental influences and stochastic events affecting the genotype–phenotype correspondence [54]. Here, we tackled this question by estimating variation in life-history traits and fitness of *A. thaliana* maternal lines from Iberian populations characterized by low and high genotypic and ecological diversity in edge and core environments, respectively. Although the large-scale effects of environmental and ecological variation on *A. thaliana*’s life-history traits are well known [23, 38, 55–58], the impact of environmental variation on within-population genetic and phenotypic variation is less understood [11, 15, 30, 44, 47, 59–61], mainly because working with populations is far more challenging and demanding than handling accessions. Hence, population-based approaches represent a fundamental piece to better grasp the species’ evolutionary ecology and its response to environmental changes.

 In this study, we first expected that *A. thaliana* populations from edge environments with low genotypic and ecological diversity would exhibit lower phenotypic variation, as directional selection in edge environments should have imposed stronger shifts in trait distribution by increasing the frequency of genotypes with eventually higher fitness [57, 62]. This process may occur particularly fast in a scenario of restricted gene flow [63, 64], such as Iberian *A. thaliana* [11]. The results, however, showed that all populations expressed a substantial amount of phenotypic variation in practically all traits in our common garden experiments (Fig. 2 and Table S2). In addition, we found consistent patterns of variation for recruitment and flowering time, as these two traits did not differ between groups of populations, had non-zero broad-sense heritability values in almost all populations and experiments (Table S3), and represented major targets of directional selection, particularly flowering time in the first experiment (Table 5 and Table S5). Hence, selection has probably operated in all populations by adjusting the sequential nature of germination and flowering phenology to their own environments [65–67], but without depleting phenotypic variability, as observed in other annuals even in extremely harsh environments [68]. A recent study on phenotypic variation in urban *A. thaliana* populations also indicated that environmental filtering in cities promoted the maintenance of phenotypic diversity in fitness-related traits [69], stressing the role of environmental heterogeneity for the preservation of genetic diversity in plants.

Based on the dramatic change in the intensity and direction of selection on flowering time between experiments, as observed elsewhere in *A. thaliana* [43, 67, 70–72], and to a much lesser extent on recruitment in the second experiment (Table 5), we believe that year-to-year fluctuations in environmental-driven selection is one of the forces accounting for the maintenance of phenotypic variation in populations. In fact, former studies showed that the consequences for life-history traits in populations with fluctuating and episodic selection pressures are highly context-dependent, leading to fluctuating selection responses and the maintenance of genetic variation within populations [73, 74], likely contributing to the evolutionary success of populations.

On top of that, recent findings on the extent of local adaptation using long-term experiments with recombinant inbred lines in *A. thaliana*, indicated that even locally adapted populations may not reach their fitness optimum in their environments due to the presence of maladaptive loci that remain in populations [75]. Furthermore, a limited number of genetic trade-offs and conditionally adaptive loci emerge as responsible for local adaptation in *A. thaliana* [75, 76] as well as in other plants [77–81]. As the eventual effects of genetic trade-offs and conditional neutrality depend on the environmental context and are subject to temporally variable selection [75], *A. thaliana* populations possess the means to maintain phenotypic variation in the long run. Despite the low gene flow and low outcrossing rates in natural *A. thaliana* populations [11] and the contribution of mutation accumulation to standing genetic variation and phenotypic variation [82], every effective recombination between two genetically and phenotypically distinct individuals suffices to yield rapid changes in phenotypic variation [83] and has the potential to create a progeny with a considerable amount of variation and plasticity in fitness-related traits, such as flowering time [84]. This property also accounts for the great amount of phenotypic variation and plasticity detected in our *A. thaliana* populations with either low or high genotypic and ecological diversity.

However, we detected slight differences between *A. thaliana* populations with low and high diversity that are worth mentioning. For example, populations with low diversity exhibited lower fecundity and fitness, traits whose variance is mostly determined by its environmental component, than populations with high diversity (Fig. 2). This result suggests that harboring low genotypic diversity might entail a stronger, albeit not critical, response of populations to environmental changes in an important fitness component, such as fecundity. Nonetheless, *A. thaliana* just needs a favorable year to boost fecundity to replenish the soil seed bank and potentially increase plant density in forthcoming years [15, 18], which may easily buffer the detrimental effects of poor years on fecundity. The long-term monitoring of one of our study populations since 2012 (MDC) supports this view [15], as it exhibited large year-to-year fluctuations in abundance but with no effects on the genetic and phenotypic composition. The dramatic changes in abundance in *A. thaliana* populations over time might account for the lack of a clear relationship between above and belowground abundance, as well as between abundance and genotypic diversity in our study populations. Furthermore, BON significantly differed from the other two populations with low diversity (AGU and CAI) for recruitment, flowering time and survival. In contrast, POB significantly differed from the other two populations with high diversity (MAR and MDC) for flowering time only. A similar picture was detected for among-population variation in phenotypic plasticity, as BON and POB stood out as the two populations differing from the other populations in their diversity groups (Fig. 3). Thus, local environmental features, irrespective of genotypic diversity, may strongly affect fitness-related traits and their plasticity, as BON (a seaside population) differed from AGU and CAI (two mountain pass populations), whilst POB (a coastal mixed forest population) differed from MAR and MDC (two continental scrubland populations) in several traits.

Our second expectation dealt with the effects of genotypic and ecological diversity on phenotypic plasticity of *A. thaliana* traits, which was supported by the positive relationship between environmental heterogeneity and phenotypic plasticity found in other plants [31, 32, 85, 86]. Our common garden experiments, carried out under unplanned benign and harsh conditions for *A. thaliana*, allowed us to estimate the response of maternal lines from each population to warmer temperatures and more seasonal precipitation (Fig. S3), which remarkably mimicked the predicted climatic trend across the Mediterranean Basin region in the near future [87]. Regardless of their genotypic and ecological diversity, we found that all populations encompassed maternal lines with low and high levels of phenotypic plasticity with significant differences between experiments in practically all populations and traits (Fig. 4 and Table 3). Other studies on phenotypic plasticity in *A. thaliana* also indicated that all life stages with no exception may exhibit a plastic behavior [88]. The potential shown by maternal lines from any population to be plastic for almost any trait aligns with field observations [89], experiments [65, 90] and models [91] indicating that a single genotype has the ability to produce mixtures of phenotypes differing in traits related to life-cycle variation in *A. thaliana*.

Given the lack of a relationship between phenotypic plasticity and fitness, we suggest a non-adaptive nature of phenotypic plasticity in this set of Iberian *A. thaliana* populations, as also found in experiments using genetic constructs [92, 93] and populations from the non-native North American range [30]. In contrast, regional-scale studies using accessions did find signs of adaptive plasticity in *A. thaliana*, either by significant relationships with fitness [43] or with the environmental variability of origin [94], suggesting that scale matters in depicting the adaptive significance of phenotypic plasticity in *A. thaliana*. Once more, the patterns that clearly emerge with accession-based approaches at a regional scale are much more difficult to detect at the population scale.

Despite the existence of maternal lines with significant variation between experiments in all populations (Fig. 4), mostly decreasing life-history traits and fitness, canalized maternal lines with non-significant differences between experiments were substantial or even dominant in all populations. We estimated that between 40 and 80% of maternal lines that completed the life cycle in the second experiment exhibited canalization across populations and traits, irrespective of their diversity group. The duality of maternal lines with a trend for plasticity and canalization coexisting within the same population is supported by theoretical work indicating that plasticity and canalization in populations are favored in epochs of environmental shifts and stability, respectively [95]. Nevertheless, the genetic and epigenetic contributions to integrated phenotypes [96] along with the degree of environmental fluctuations may determine the extent of plasticity and canalization. In fact, in *A. thaliana*, we know that specific allele combinations of seed dormancy and/or flowering time genes determine key developmental traits as well as their phenotypic plasticity [84, 92, 93, 97, 98] and that the diversity of parental environments modulates phenotypic modifications in the offspring for a few generations [35, 88, 99] through environmentally-induced epigenetic changes [100, 101].

## Conclusions

Natural *A. thaliana* populations that have remained undisturbed for long time are able to maintain phenotypic variation and phenotypic plasticity for life-history traits with an important contribution to fitness. Edge and core environments with low and high ecological diversity, respectively, may influence the genotypic composition of *A. thaliana* populations, probably through effective recombination from very different outcrossing rates between edge and core environments (Table 1), but seem not to determine their phenotypic diversity in the response of genotypes to the idiosyncrasy of each environment. Beyond the level of genetic variation harbored by a population, maintaining canalized and sensitive phenotypes to environmental fluctuations within populations represents an extraordinary guarantee to endure in changing environments, but only demanding within-population approaches have the power to depict such patterns.

### Supplementary Information

**Supplementary Material 1. **

## Data Availability

Data deposited in the Dryad repository: 10.5061/dryad.98sf7m0qp [102]. Seeds are publicly available through the Nottingham Arabidopsis Stock Centre (NASC; http://arabidopsis.info). Raw whole-genome sequences are available at NCBI SRA (BioProject number PRJNA998580; https://www.ncbi.nlm.nih.gov/bioproject/PRJNA998580). Field sampling was conducted in locations where permission was not required, except at BON that is located within Doñana Natural Area (permission issued by Estación Biológica de Doñana on March 3, 2016; Ref.: 20151073000000570). Based on the Royal Decree of the Spanish legislation (Real Decreto 124/2017, de 24 de febrero; https://www.boe.es/eli/es/rd/2017/02/24/124), the genetic resources included in this study fall within the definition of “taxonomic purposes”.

## Abbreviations

DNA: Deoxyribonucleic acid
GPS: Global positioning system
RDPI: Relative distance plasticity index
REML: Restricted maximum likelihood
SNP: Single nucleotide polymorphism
SSR: Simple sequence repeats
VCF: Variant call format
WGS: Whole-genome sequence
