# Supplementary material for: Variation and plasticity in life-history traits and fitness of wild Arabidopsis thaliana populations are not related to their genotypic and ecological diversity
Source: BMC Ecol Evol. 2024 May 3;24:56. doi: 10.1186/s12862-024-02246-x (PMC11067129; doi:10.1186/s12862-024-02246-x)
Supplement: Supplementary file 1 — Supplementary Material 1. [file 12862_2024_2246_MOESM1_ESM.docx]

**Supplementary Methods**

**Methodological caveat: sampling problems at BON**

In BON, *A. thaliana* behaves differently than in the rest of populations, as individuals are generally small and the number of ripe fruits shedding seed is limited to one or two per individual at a time. This is because *A. thaliana*’s life cycle at BON is very short, germinating in December/January and shedding seed in February/March (F.X. Picó, pers. obs.). We do not see this pattern in the rest of study populations, which are probably made of combinations of autumn- and spring-germinated cohorts, as observed elsewhere [1, 2]. In March 2017, a heavy rain wiped out the BON population when most of the individuals were ready for seed collection. As a result, 14 of 45 maternal lines from BON came from the 2017 sampling, whereas the rest came from a sampling conducted a year later in March 2018. Seeds collected in 2018 were multiplied in summer 2018 prior to the first experiment. Preliminary analysis indicated that maternal lines collected in 2018 had germinating fractions lower than those collected in 2017 in the first experiment (mean±SE germination fraction of maternal lines collected in 2017 and 2018 = 0.72±0.01 and 0.25±0.03). These differences disappeared in the second experiment (0.71±0.01 and 0.73±0.01), indicating that seeds from maternal lines collected in March 2018 had not completed the after-ripening after the multiplication experiment. Thus, we excluded maternal lines from BON collected in 2018 from all analyses including the first experiment. Overall, although this problem implied some limitations in some analyses (e.g. phenotypic plasticity, selection analysis), full data including the 45 maternal lines from the second experiment indicated that the 14 maternal lines from the first experiment did not render biased results for BON (Fig. 2 and Table 4).

**Methodological caveat: sampling problems in the second common garden experiment**

Spain was in national lockdown due to the coronavirus pandemic between mid-March and mid-June 2020, which rendered us unable to monitor the experiment until June 17, 2020, when we collected all fruiting plants from those maternal lines that did not flower or did not finish fruiting before our last visit on March 11, 2020. By the time of lockdown, we had assigned all flowering times and collected all fruiting plants from practically all replicates and maternal lines from BON, MAR, and MDC (Table S1). In contrast, 2, 8, and 22 maternal lines from POB, CAI, and AGU, respectively, had no flowering time assigned (Table S1). As we set up replicates of both experiments in the very same stands of the experimental facility (Fig. S2) and flowering time in the second experiment systematically took place a few days earlier than in the first experiment in all maternal lines, we assigned flowering time dates to those replicates with fruiting plants but no flowering date. Based on the estimated average differences between replicates of maternal lines from each population between experiments, we imputed the same difference to replicates with missing flowering dates. For maternal lines with no replicates with flowering dates, we used mean differences among maternal lines within each population to impute flowering dates. Overall, we assigned flowering dates to 11, 21, and 38% of replicates in POB, CAI, and AGU, respectively (Table S1). Analyses with and without imputation yielded consistent conclusions (results not shown).

**Whole-genome shot-gun sequencing**

We obtained genome sequences from *A. thaliana* maternal lines from the six populations by generating paired-end libraries obtained at the Gregor Mendel Institute (Vienna Biocenter, Vienna, AT). DNA was isolated from 14-day-old rosette leaves using NucleoMag Plant kit for DNA purification (Macherey-Nagel, Düren, DE). Tagmentation-based libraries were prepared from 3 ng of DNA per sample with in-house Tn5 transposase. Libraries were enriched by 10 cycles of PCR amplification. Illumina Nextera DNA indexes (i7/i5) were used to allow multiplexing. Size selection and PCR clean-up were performed with in-house SPRI beads. Libraries were validated by Fragment Analyzer™ Automated CE System (Advanced Analytical Technologies, Santa Clara, US) and pooled in equimolar concentration. Libraries were sequenced on an Illumina NovaSeq analyzer (Illumina, San Diego, US) using manufacturer’s standard cluster generation and sequencing tools. Raw sequences of this study are available at NCBI SRA under the BioProject number PRJNA998580.

We analyzed sequences at the Bioinformatics for Genomics and Proteomics Service of the Centro Nacional de Biotecnología (Madrid, ES). SNP calling and genotyping was carried out following the pipelines described in [3]. In brief, sample quality was tested with FastQC tool v.0.11.5 (http://www.bioinformatics.babraham.ac.uk/projects/fastqc) and raw reads were filtered with Cutadapt software v.1.15 [4]. We discarded reads with an average quality lower than 20 or with a length smaller than 50. Sequences from all maternal lines were aligned against Columbia reference strain (TAIR10) with BWA software v.0.7.17 [5]. PCR duplicates were removed with Samtools v.1.6 [6]. Sequencing depths (average sequencing depth = 23.7x ± 8.5) and coverages of the nuclear genome (mean coverage = 89%; at least 3 reads) were calculated with bcftools v.1.6 [7].

We used bcftools for variant calling on each maternal line alignment. Indels were filtered out to maintain only SNPs in the variant call format (VCF) files [8]. SNPs were genotyped only at genomic positions with a minimum depth of 3 reads. All maternal line variant files were then merged with bcftools into a single multi-sample VCF file containing 5.5 M SNPs. This file was filtered to remove low quality SNPs, keeping 3.5 M binary SNPs displaying two ACTG classes, with heterozygosity lower than 3% and corresponding to the two alternative ACTG classes detected in that position, and at least 55% non-missing genotyped maternal lines. Heterozygous calls in these SNPs were rescored to the major frequency allele. We thus generated a final VCF file containing 3 515 491 nuclear SNPs genotyped in 298 of 306 maternal lines with an average missing information per SNP of 11.9%. We discarded one maternal line with less than 10x depth and less than 30% genome coverage with 3 reads, and seven more with an average heterozygosity higher than 30%.

Importantly for this study, we generated a pairwise matrix of allele differences from this VCF file, which was used to identify pairs of maternal lines with nearly identical genotypes. Pairs of samples with genetic distances lower than 0.001 were considered as carrying the same genotype, because this was the genotyping error we estimated by sequencing twice five MDC samples and in agreement with error sequencing rates described for Illumina short reads [9].

**References**

[1] Montesinos A, Tonsor ST, Alonso-Blanco C, Picó FX. Demographic and genetic patterns of variation among populations of *Arabidopsis thaliana* from contrasting native environments. PLoS One. 2009;4:e7213. <https://doi.org/10.1371/journal.pone.0007213>.

[2] Picó FX. Demographic fate of *Arabidopsis thaliana* cohorts of autumn- and spring-germinated plants along an altitudinal gradient. J Ecol. 2012;100:1009-18. <https://doi.org/10.1111/j.1365-2745.2012.01979.x>.

[3] Arteaga N, Savic M, Méndez-Vigo B, Fuster-Pons A, Torres-Pérez R, Oliveros JC, et al. MYB transcription factors drive evolutionary innovations in Arabidopsis fruit trichome patterning. Plant Cell. 2021;33:548-65. <https://doi.org/10.1093/plcell/koaa041>.

[4] Martin M. Cutadapt removes adapter sequences from high-throughput sequencing reads. EMBnet J. 2011;17:10-2. <https://doi.org/10.14806/ej.17.1.200>.

[5] Li H, Durbin R. Fast and accurate long-read alignment with Burrows-Wheeler transform. Bioinformatics. 2010;26:589-95. <https://doi.org/10.1093/bioinformatics/btp698>.

[6] Li H, Handsaker B, Wysoker A, Fennell T, Ruan J, Homer N, Marth G, et al. The sequence alignment/map format and SAMtools. Bioinformatics. 2009;25:2078-79. <https://doi.org/10.1093/bioinformatics/btp352>.

[7] Li H. A statistical framework for snp calling, mutation discovery, association mapping and population genetical parameter estimation from sequencing data. Bioinformatics. 2011;27:2987-93. <https://doi.org/10.1093/bioinformatics/btr509>.

[8] Danecek P, Auton A, Abecasis G, Albers CA, Banks F, DePristo MA, et al. The variant call format and VCFtools. Bioinformatics. 2011;27:2156-58. <https://doi.org/10.1093/bioinformatics/btr330>.

[9] Stoler N, Nekrutenko A. Sequencing error profiles of Illumina sequencing instruments. NAR Genom. Bioinformatics. 2021;27:lqab019. <https://doi.org/10.1093/nargab/lqab019>.

**Table S1** Imputation of missing flowering dates in the second experiment (2019-2020) for each *A. thaliana* population with low and high genotypic and ecological diversity in edge and core environments, respectively. The number and percentage of maternal lines with no replicates with flowering time assigned that had to be imputed is given. The total number and percentage of replicates without flowering time assigned that had to be imputed is also indicated.

| Population | No. maternal lines  (missing/total) | % Imputed maternal lines | No. replicates (missing/total) | % Imputed replicates |
| --- | --- | --- | --- | --- |
| AGU (low) | 22/55 | 40.00 | 169/440 | 38.41 |
| BON (low) | 0/45 | 0.00 | 0/360 | 0.00 |
| CAI (low) | 8/50 | 16.00 | 84/400 | 21.00 |
| MAR (high) | 0/50 | 0.00 | 3/400 | 0.75 |
| MDC (high) | 0/53 | 0.00 | 3/424 | 0.71 |
| POB (high) | 2/53 | 3.77 | 47/424 | 11.08 |

**Table S2**. Coefficients of variation (CV) for life-history traits and fitness of *A. thaliana* populations with low (AGU, BON, and CAI) and high (MAR, MDC, and POB) genotypic and ecological diversity in edge and core environments, respectively, estimated in two common garden experiments. CV values are shown for the first and second experiments and for phenotypic plasticity between the two experiments. Sample sizes are shown in parenthesis. For each trait, minimum and maximum CV values are indicated in boldface.

First experiment (2018-2019)

| Trait | AGU  (low; 55) | BON  (low; 14) | CAI  (low; 50) | MAR (high; 50) | MDC (high; 53) | POB  (high; 53) |
| --- | --- | --- | --- | --- | --- | --- |
| Recruitment | **21.34** | **7.44** | 15.86 | 17.46 | 15.52 | 17.79 |
| Flowering time | 3.42 | **1.76** | 2.90 | 3.80 | 2.69 | **4.49** |
| Survival | 11.87 | **4.41** | **12.05** | 7.59 | 7.99 | 9.99 |
| Fecundity | 23.60 | **23.35** | 26.66 | 30.04 | 26.93 | **31.11** |
| Fitness | 28.65 | **24.13** | **32.39** | 30.96 | 29.34 | 29.45 |

Second experiment (2019-2020)

| Trait | AGU  (low; 52) | BON  (low; 42) | CAI  (low; 35) | MAR (high; 47) | MDC (high; 48) | POB  (high; 51) |
| --- | --- | --- | --- | --- | --- | --- |
| Recruitment | 29.63 | **9.19** | **53.22** | 37.84 | 37.51 | 22.71 |
| Flowering time | 3.00 | **1.99** | 2.44 | 4.98 | 4.17 | **5.10** |
| Survival | 27.33 | **18.09** | **21.07** | 24.68 | 22.23 | 22.65 |
| Fecundity | 41.94 | 34.41 | **62.94** | 40.33 | 37.86 | **29.79** |
| Fitness | 55.81 | **38.41** | **60.60** | 43.15 | 57.55 | 38.92 |

Phenotypic plasticity between experiments

| Trait | AGU  (low; 45) | BON  (low; 14) | CAI  (low; 35) | MAR (high; 47) | MDC (high; 48) | POB  (high; 51) |
| --- | --- | --- | --- | --- | --- | --- |
| Recruitment | 73.61 | 65.89 | **59.14** | 93.48 | **102.28** | 80.76 |
| Flowering time | 68.16 | **43.19** | 63.12 | 59.55 | 68.91 | **83.14** |
| Survival | 60.00 | 59.72 | **67.49** | 54.02 | **46.59** | 52.08 |
| Fecundity | 57.27 | 54.97 | 55.74 | **71.97** | 66.65 | **49.61** |
| Fitness | 70.50 | **39.12** | 73.75 | 70.11 | 63.66 | **71.10** |

**Table S3**. Broad-sense heritability (*H^2^*) for life-history traits and fitness of *A. thaliana* populations with low (AGU, BON, and CAI) and high (MAR, MDC, and POB) genotypic and ecological diversity in edge and core environments, respectively, for the first (2018-2019) and second (2019-2020) common garden experiment. Mean (95% CI) values are given for each trait, population, and experiment. All *H^2^* values significantly different from zero are in boldface. Sample size for each population and experiment used in the analysis are given in parenthesis.

First experiment (2018-2019)

| Trait | AGU (low; 55) | BON (low; 14) | CAI (low; 50) | MAR (high; 50) | MDC (high; 53) | POB (high; 53) |
| --- | --- | --- | --- | --- | --- | --- |
| Recruitment | **0.26 (0.16–0.32)** | 0.00 (0.00–0.00) | 0.08 (0.00–0.13) | **0.16 (0.06–0.21)** | **0.09 (0.01–0.14)** | **0.19 (0.09–0.25)** |
| Flowering time | **0.56 (0.46–0.61)** | 0.07 (0.00–0.24) | **0.52 (0.41–0.57)** | **0.63 (0.53–0.67)** | **0.39 (0.28–0.44)** | **0.70 (0.62–0.74)** |
| Survival | 0.02 (0.00–0.07) | 0.00 (0.00–0.00) | **0.09 (0.01–0.15)** | 0.01 (0.00–0.06) | 0.00 (0.00–0.05) | 0.05 (0.00–0.11) |
| Fecundity | 0.00 (0.00–0.05) | 0.08 (0.00–0.16) | 0.01 (0.00–0.06) | 0.12 (0.03–0.17) | 0.06 (0.00-0.11) | 0.09 (0.00–0.15) |
| Fitness | 0.02 (0.00–0.07) | 0.06 (0.00–0.14) | 0.05 (0.00–0.10) | 0.11 (0.02–0.17) | 0.07 (0.00–0.12) | 0.05 (0-00–0.10) |

Second experiment (2019-2020)

| Trait | AGU (low; 52) | BON (low; 42) | CAI (low; 35) | MAR (high; 47) | MDC (high; 48) | POB (high; 51) |
| --- | --- | --- | --- | --- | --- | --- |
| Recruitment | **0.38 (0.26–0.43)** | 0.06 (0.00–0.12) | **0.48 (0.32–0.53)** | **0.66 (0.56–0.69)** | **0.51 (0.39–0.55)** | **0.42 (0.35–0.45)** |
| Flowering time | **0.64 (0.54–0.67)** | 0.08 (0.00–0.13) | **0.28 (0.10–0.34)** | **0.65 (0.55–0.69)** | **0.46 (0.33–0.50)** | **0.72 (0.64–0.74)** |
| Survival | 0.10 (0.00–0.17) | 0.09 (0.00–0.15) | 0.08 (0.00–0.15) | 0.02 (0.00–0.08) | 0.00 (0.00–0.00) | 0.00 (0.00–0.00) |
| Fecundity | **0.20 (0.07–0.26)** | 0.00 (0.00–0.00) | 0.12 (0.00–0.20) | 0.04 (0.00–0.11) | 0.06 (0.00–0.13) | 0.02 (0.00–0.09) |
| Fitness | **0.17 (0.04–0.23)** | 0.00 (0.00–0.00) | 0.07 (0.00–0.15) | 0.00 (0.00–0.00) | **0.15 (0.01–0.22)** | 0.00 (0.00–0.00) |

**Table S4**. Correlations between pairs of life-history traits and fitness in *A. thaliana* from maternal lines from populations with low (AGU, BON, and CAI) and with high (MAR, MDC, and POB) genotypic and ecological diversity in edge and core experiment, respectively, estimated from the first (2018-2019) and second (2019-2020) common garden experiment. Sample size for each population and experiment used in the analysis is given in parenthesis. Correlation coefficients for each population, experiment, and pairwise comparison are given. Asterisks indicate significance estimated with Dutilleul’s *t*-test: ***; *P* <0.001, **; *P* <0.01, *; *P* <0.05, *ns*; non-significant.

First experiment (2018-2019)

| Traits | AGU (50) | BON (14) | CAI (50) | MAR (50) | MDC (53) | POB (53) |
| --- | --- | --- | --- | --- | --- | --- |
| Recruitment *vs*. Flowering time | -0.086 *ns* | -0.309 *ns* | -0.127 *ns* | **-0.379 **** | **-0.352 **** | **-0.340 *** |
| Recruitment *vs*. Survival | 0.236 *ns* | **0.618 *** | **0.348 *** | **0.525 ***** | **0.367 **** | 0.151 *ns* |
| Recruitment *vs*. Fecundity | -0.021 *ns* | 0.367 *ns* | -0.007 *ns* | **0.305 *** | 0.162 *ns* | 0.159 *ns* |
| Recruitment *vs*. Fitness | 0.102 *ns* | 0.475 *ns* | 0.149 *ns* | **0.366 **** | 0.242 *ns* | 0.256 *ns* |
| Flowering time *vs*. Survival | **-0.358 **** | -0.273 *ns* | **-0.493 **** | **-0.476 **** | -0.035 *ns* | -0.228 *ns* |
| Flowering time *vs*. Fecundity | **-0.531 *** | -0.224 *ns* | **-0.518 **** | **-0.447 ***** | **-0.512 ***** | **-0.518 ***** |
| Flowering time *vs*. Fitness | **-0.555 ***** | -0.247 *ns* | **-0.568 **** | **-0.545 ***** | **-0.525 ***** | **-0.559 ***** |
| Survival *vs*. Fecundity | 0.233 *ns* | 0.055 *ns* | **0.488 ***** | 0.260 *ns* | 0.095 *ns* | -0.076 *ns* |

**Table S4**. Continued.

Second experiment (2019-2020)

| Traits | AGU (48) | BON (41) | CAI (35) | MAR (47) | MDC (48) | POB (51) |
| --- | --- | --- | --- | --- | --- | --- |
| Recruitment *vs*. Flowering time | -0.103 *ns* | -0.161 *ns* | -0.055 *ns* | -**0.590 ***** | -0.160 *ns* | **-0.346 *** |
| Recruitment *vs*. Survival | -0.203 *ns* | **0.606 ***** | -0.149 *ns* | 0.122 *ns* | 0.125 *ns* | **0.320 *** |
| Recruitment *vs*. Fecundity | **-0.388 *** | 0.154 *ns* | **-0.486 **** | **-0.495 **** | -0.187 *ns* | 0.054 *ns* |
| Recruitment *vs*. Fitness | **-0.347 *** | **0.368 *** | **-0.446 *** | **-0.452 **** | -0.005 *ns* | 0.168 *ns* |
| Flowering time *vs*. Survival | 0.110 *ns* | **-0.339 *** | -0.230 *ns* | **-0.352 *** | **-0.411 **** | 0.081 *ns* |
| Flowering time *vs*. Fecundity | 0.194 *ns* | -0.036 *ns* | -0.069 *ns* | **0.524 **** | 0.044 *ns* | 0.132 *ns* |
| Flowering time *vs*. Fitness | 0.142 *ns* | -0.232 *ns* | -0.147 *ns* | 0.328 *ns* | -0.164 *ns* | 0.106 *ns* |
| Survival *vs*. Fecundity | **0.315 *** | -0.053 *ns* | 0.203 *ns* | -0.087 *ns* | 0.164 *ns* | 0.171 *ns* |

**Table S5**. Linear and quadratic selection gradients (*β* and *γ*) and selection differentials (*s* and *C*) for recruitment and flowering time for *A. thaliana* populations with low (AGU, BON, and CAI) and with high (MAR, MDC, and POB) genotypic and ecological diversity in edge and core environments, respectively, estimated from the first (2018-2019) and second (2019-2020) common garden experiment. Sample size for each population and experiment used in the analysis is given in parenthesis. Selection gradients and selection differentials for BON in the first experiment could not be estimated due to low sample size. Significance: ∗∗∗, *P* <0.0001; ∗∗, *P* <0.01; ∗, *P* <0.05; *ns*, non-significant.

First experiment (2018-2019)

|  |  | Linear |  |  | Quadratic |  |  |
| --- | --- | --- | --- | --- | --- | --- | --- |
| Population |  | Recruitment | Flowering time |  | Recruitment | Flowering time | Interaction |
| AGU (low; 55) | *β* | 0.028 (0.044) *ns* | -0.219 (0.077) ** | *γ* | 0.098 (0.091) *ns* | -0.145 (0.310) *ns* | -0.086 (0.090) *ns* |
|  | *s* | 0.014 (0.035) *ns* | -0.149 (0.034) *** | *C* | 0.056 (0.035) *ns* | 0.148 (0.060) ** | -0.074 (0.041) *ns* |
| BON (low; 14) | *β* | – | – | *γ* | – | – | – |
|  | *s* | – | – | *C* | – | – | – |
| CAI (low; 50) | *β* | 0.006 (0.045) *ns* | -0.217 (0.057) *** | *γ* | -0.056 (0.073) *ns* | 0.069 (0.101) *ns* | 0.053 (0.067) *ns* |
|  | *s* | 0.048 (0.039) *ns* | -0.181 (0.035) *** | *C* | -0.019 (0.055) *ns* | -0.106 (0.064) *ns* | -0.016 (0.041) *ns* |
| MAR (high; 50) | *β* | 0.045 (0.050) *ns* | -0.151 (0.058) * | *γ* | -0.045 (0.110) *ns* | 0.103 (0.146) *ns* | -0.010 (0.101) *ns* |
|  | *s* | 0.111 (0.036) ** | -0.165 (0.038) *** | *C* | -0.077 (0.051) *ns* | 0.044 (0.046) *ns* | -0.016 (0.039) *ns* |
| MDC (high; 53) | *β* | 0.016 (0.039) *ns* | -0.179 (0.039) *** | *γ* | 0.021 (0.074) *ns* | 0.033 (0.070) *ns* | 0.010 (0.053) *ns* |
|  | *s* | 0.070 (0.043) *ns* | -0.151 (0.038) *** | *C* | 0.004 (0.059) *ns* | 0.014 (0.057) *ns* | -0.017 (0.050) *ns* |
| POB (high; 53) | *β* | -0.005 (0.040) *ns* | -0.216 (0.043) *** | *γ* | 0.007 (0.084) *ns* | -0.099 (0.116) *ns* | -0.008 (0.052) *ns* |
|  | *s* | 0.074 (0.035) * | -0.173 (0.035) *** | *C* | 0.015 (0.041) *ns* | 0.036 (0.069) *ns* | -0.028 (0.034) *ns* |

**Table S5**. Continued.

Second experiment (2019-2020)

|  |  | Linear |  |  | Quadratic |  |  |
| --- | --- | --- | --- | --- | --- | --- | --- |
| Population |  | Recruitment | Flowering time |  | Recruitment | Flowering time | Interaction |
| AGU (low; 52) | *β* | -0.115 (0.083) *ns* | 0.109 (0.142) *ns* | *γ* | 0.132 (0.200) *ns* | 0.174 (0.362) *ns* | -0.111 (0.149) *ns* |
|  | *s* | -0.189 (0.100) *ns* | 0.089 (0.071) *ns* | *C* | 0.258 (0.200) *ns* | -0.031 (0.084) *ns* | -0.041 (0.109) *ns* |
| BON (low; 42) | *β* | 0.108 (0.084) *ns* | -0.079 (0.094) *ns* | *γ* | -0.086 (0.187) *ns* | -0.194 (0.185) *ns* | -0.034 (0.126) *ns* |
|  | *s* | 0.134 (0.050) *ns* | -0.089 (0.042) *ns* | *C* | -0.100 (0.059) *ns* | -0.183 (0.094) *ns* | 0.001 (0.049) *ns* |
| CAI (low; 35) | *β* | -0.249 (0.128) * | 0.014 (0.143) *ns* | *γ* | 0.123 (0.370) *ns* | -0.256 (0.299) *ns* | -0.258 (0.236) *ns* |
|  | *s* | -0.263 (0.090) ** | -0.087 (0.074) *ns* | *C* | 0.064 (0.111) *ns* | -0.194 (0.085) * | -0.117 (0.060) * |
| MAR (high; 47) | *β* | -0.159 (0.095) *ns* | 0.054 (0.096) *ns* | *γ* | 0.073 (0.233) *ns* | -0.080 (0.281) *ns* | -0.054 (0.139) *ns* |
|  | *s* | -0.191 (0.048) *** | 0.138 (0.053) * | *C* | 0.053 (0.063) *ns* | -0.008 (0.055) *ns* | -0.032 (0.050) *ns* |
| MDC (high; 48) | *β* | 0.006 (0.109) *ns* | -0.057 (0.113) *ns* | *γ* | -0.017 (0.269) *ns* | -0.123 (0.317) *ns* | -0.054 (0.142) *ns* |
|  | *s* | -0.003 (0.074) *ns* | -0.092 (0.072) *ns* | *C* | 0.067 (0.114) *ns* | 0.037 (0.095) *ns* | -0.050 (0.081) *ns* |
| POB (high; 51) | *β* | 0.029 (0.077) *ns* | 0.169 (0.080) * | *γ* | -0.093 (0.164) *ns* | 0.395 (0.261) *ns* | -0.172 (0.100) *ns* |
|  | *s* | 0.064 (0.056) *ns* | 0.040 (0.056) *ns* | *C* | -0.041 (0.094) *ns* | 0.080 (0.064) *ns* | -0.020 (0.041) *ns* |

**Figure S1**. Photographs and information of each *A. thaliana* populations with low (AGU, BON, and CAI) and high (MAR, MDC, and POB) genotypic and ecological diversity in edge and core environments, respectively. Information includes population name and province, geographic coordinates, altitude, annual mean minimum and maximum temperatures, annual total precipitation, population area, percentage of area occupied by vegetation types, and regional-scale suitability area around each population (50 km radius).

**Figure S1.** Continued.

**Figure S2**. Photographs of the experimental facility of El Castillejo Botanical Garden. Numbers indicate the position of the blocks in both experiments.

**Figure S3**. Daily minimum temperature, maximum temperature and precipitation over the two experiments conducted at the experimental facility of El Castillejo Botanical Garden. Both experiments were established on November 15 (2018 and 2019) and terminated on April 15 (2019 and 2020).

**Figure S4**. Voronoi diagrams depicting suitability for *A. thaliana* in populations with low (upper maps: AGU, BON, and CAI) and high (lower maps: MAR, MDC, and POB) genotypic and ecological diversity in edge and core environments, respectively. Red dots indicate the location of aboveground spots in which *A. thaliana* abundance was estimated in two years (between early March and early May of 2017 and 2018).

**Figure S5**. Number of unique (represented by one maternal line only) and non-unique genotypes from each *A. thaliana* populations with low (AGU, BON, and CAI) and high (MAR, MDC, and POB) genotypic and ecological diversity in edge and core environments, respectively. For each population, each genotype is represented by a different color and box size is proportional to the number of maternal lines found within each genotype. For unique genotypes, numbers indicate the total number of maternal lines with unique genotypes in each population. The number of maternal lines with significant (*s*) and non-significant (*ns*) variation in traits between experiments are indicated for each genotype and population.
